# Medical Association of Georgia

**Published:** 1867-05

**Authors:** L. H. Orme

**Affiliations:** Rec. Sec.


### MEDICAL ASSOCIATION OF THE STATE OF GEORGIA.

Convened in Griffin, at the M. E. Church, on the morning of the 10th of April, 1867.

The meeting having been called by the President, Dr. Means, the Rev. Mr. Winn was introduced, and opened the proceedings with prayer; after which, Col. A. D. Nunnally, Alderman, on behalf of the city, welcomed the members to Griffin.

Upon the call of the roll, the following members responded:—

A. Means, Oxford; L. H. Orme, Atlanta; R. C. Word, Atlanta; T. S. Powell, Atlanta; G. G. Crawford, Atlanta; John D. Fish, Savannah; J. H. Connally, Griffin; G. M. McDowel, Barnesville; W. C. Asher, Atlanta; C. L. Redwine, Atlanta; E. L. Connally, Albany; E. J. Roach, Atlanta; W. C. More, Atlanta; Chas. Pinckney, Atlanta; W. M. Charters, Savannah; J. M. Boring, Atlanta; A. W. Griggs, West-Point; W. S. Armstrong, Atlanta; N. B. Drewry, Griffin; E. F. Knott, Griffin; J. T. Banks, Griffin; Jno. L. Moore, Griffin; T. M. Darnall, Griffin; W. F. Westmoreland, Atlanta; DeSaussure Ford, Augusta; L. J. Dupree, Domilla; J. H. M. Barrett, Griffin; J. F. Alexander, Atlanta; J. N. Simmons, Atlanta.

Upon motion of Dr. J. T. Banks, the order of business was suspended for the admission of new members. The following named physicians were presented, vouched for, and elected members of the Association:—

Ed S. Ray, Atlanta; E. Geddings, Augusta; W. T. Holt, Macon; F. G. Castlin, Macon; J. J. Knott, Griffin; Geo. B. Beecher, Griffin; J. A. Davis, Atlanta; A. Hunnicutt, Newnan; J. P. Touchstone, Newton; Thos. Mitchell, Griffin; J. D. Yarber, Griffin; W. H. Touchstone, Griffin; E. A. Flewellen, Thomaston; M. J. Daniel, Griffin; J. G. Thomas, Savannah; R. V. Reid Zebulon; T. Heard, Griffin; R. P. Myers, Savannah; L. L. Strozier, Albany.

Rules were, upon motion, again suspended for the purpose of inviting Mayor and Aldermen and members of the press to seats on the floor.

The proceedings of last meeting were then read and adopted.

Upon motion of Dr. J. T. Banks, the session adjourned until 2½ o'clock, P. M.

2½ O'Clock, P. M.

Meeting called to order by the President. Minutes read and adopted.

An election for officers of the Association for the ensuing year was then held, which resulted as follows:—

Dr. W. M. Charters, of Savannah, President; Dr. T. S. Powell, of Atlanta, 1st Vice President; Dr. DeSaussure Ford, of Augusta, 2d Vice President; Dr. L. H. Orme, Recording Sec'y; Dr. R. P. Myers, of Savannah, Corresponding Secretary; Dr. John D. Fish, of Savannah, Treasurer.

On motion, Dr. E. L. Connally, of Albany, Dr. W. F. Westmoreland, and J. T. Banks were appointed a committee to conduct the newly elected President to the chair.

Dr. A. Means, before retiring from the chair, addressed the Association. He congratulated it upon its choice in the selection of his successor in office. It would probably be the last time that he would serve them in the capacity of presiding officer. He had prepared no address, but would send a message to those rising up to stand upon the stage that he was leaving.

He then dwelt for some time with power and eloquence upon the subject of electricity in its connection with physiology and pathology, and the invaluable contributions which it is destined to supply to the resources of the medical profession, and took an affecting leave of the body.

The newly elected President, Dr. Charters, then, upon assuming the duties of his office, addressed the Association in an appropriate manner.

The following resolutions were then introduced by Dr. L. H. Orme, and after some debate, were postponed until morning session:—

Whereas, According to article 1, Code of Ethics of the American Medical Association, "every individual, on entering the profession, as he becomes entitled to all its privileges and immunities, incurs an obligation to exert his best abilities to maintain its dignity and honor," and "to exalt its standing;" and

Whereas, According to article 4, of said Code of Ethics, "a *regular* medical education furnishes the *only* presumptive evidence of professional abilities and acquirements, and ought to be the *only acknowledged right* of an individual to the exercise and honors of his profession;" therefore—

*Resolved*, That while the fact is recognized that there are in our midst medical practitioners worthy, talented and useful, who, from lack of means or other cause, have failed to obtain a diplima; yet, as the earning of the degree of doctor of medicine furnishes the only presumptive evidence of a *regular* medical education, the Georgia Medical Association, fully alive to the honor, dignity, and true interests of the profession, deems the admission, *in future*, of non-graduates to membership, a violation of the spirit which governs the code of medical ethics.

*Resolved*, That *hereafter*, no individual shall be entitled to membership in this Association who has not received the degree of doctor of medicine from some medical school of known and acknowledged respectability, and as such recognized by the American Medical Association.

*Resolved*, That the portion of the constitution which provides for the admission to membership in the Georgia Medical Association of State licentiates be stricken out.

On motion, meeting adjourned until 9 o'clock, A. M., April 11th.

---

April 11th, 9 O'Clock, A. M.

Meeting called to order by the President.

Minutes read and adopted.

Resolutions by Dr. L. H. Orme, the day previous, were then brought up, and after some discussion, were adopted.

Reports from auxiliary societies were then called for. The following societies then reported:

Georgia Medical Society, Savannah, Ga.

Atlanta Medical Society, Atlanta, Ga.

Georgia and Alabama Med. Association, West Point, Ga.

Fulton County Medical Society, Atlanta, Ga.

Upon written communications being called for, Dr. R. C. Word, of Atlanta, read before the Association, an able essay upon the duties of the people to the medical profession; after which the following resolution was offered by Dr. T. S. Powell, and adopted:

*Resolved*, 1. That the thanks of this Association are due to Dr. R. C. Word for the truthful and able essay he has read on the obligations of the people to the medical profession.

*Resolved*, 2. That this Association fully endorse the sentiments therein contained, and recommend that the essay be published in the Medical Journals of the State.

Upon motion of Dr. Roach, the order of business was suspended, in order to elect a place for the next annual meeting.

Upon motion of Dr. Crawford, the choice was ballotted for, and resulted in the selection of Augusta.

On motion of Dr. Alex. Means, the rules were then suspended, in order that he might direct the attention of the Association to a financial question in reference to the publication of Dr. Gaillard's prize essay.

Dr. W. P. Holt, of Macon, read before the Association the following report:

*Persistent Eclamsia: forced delivery accomplished by bilateral incision of the Cervix Uteri.* By Wm. P. Holt, M. D., Macon, Ga.

I was summoned at 6 P. M., on March 9th, 1867, to see Mrs.———, a *primipara*, aet. 19, stout, robust and very plethoric; was informed by her that she had "grinding" pains at regular intervals of fifteen or twenty minutes, since 11 A. M., and that she was under the impression that she was not at full term. I made an examination and found the os very high up, *undilated* and rigid. She states that she has had an unusual *fullness* about her head for the past three weeks; that her limbs were swollen, and that the pain in her arms was so great at night, as to prevent her from sleeping; that about ten days since, while going down the steps, she fell, but experienced no inconvenienc from the fall; not even soreness.

The pains continued to increase in severity; and at 10 P. M., she had heavy bearing down pains, when I again made an examination, and found the os in the *same position and condition.* Up to this time there was nothing unusual in her appearance. Expressing a desire to empty the bladder, I retired from the room, leaving her in charge of her husband and nurse, and was informed upon my return to her chamber, that she had voided urine, and had sat up before the fire warming her feet, (which, notwithstanding the application of hot irons, continued cold) and that while up, that there was a wild expression about her eyes. I detected a slight twitching about the facial muscles, and at once bled her sixteen or twenty ounces, and dispatched a messenger for my friend, Dr. D. W. Hammond.

He arrived at 12 M., when she had a violent convulsion. Upon consultation we determined to apply mustard sinapism to her extremities, cold to her head, and to try the effect of chloroform, the *free inhalation* of which seemed to modify, but not to arrest the convulsions. Examination revealed no change in the condition of the os; ordered the following:

℞.—Ol. ricini ℥ i., ol. terebinthinæ ʒ i., which she retained, though she had been *vomiting* occasionally all night. Dr. H. and myself both remained with her until 7 A. M., when I was summoned home, leaving her in his charge, and promising to return at 8½ o'clock, and to invite Dr. C. B. Nottingham to see her with us. I returned at the specified time, and was informed by the Doctor that during my absence she had thirteen (13) convulsions, and that each one was more terrific than its predecessor; that her face bacame livid, breathing stertorous; in fact, she was almost comatose. We determined to make an effort to *force* the finger through the os, and if possible, rupture the membranes, which was accomplished after much difficulty, the contraction being so firm that it was almost impossible to move the finger.

At 9 A. M., Dr. Nottingham arrived, when the patient had another convulsion, which seemed as if it would terminate her existence. Upon examination, (there being no change in the condition of the os) we determined to bleed her again, which I did *copiously*, and to wait until 12 M. for further developments, at which time Drs. H. and N. were to see her. After the loss of blood, she was more quiet, although her breathing was heavy and labored. At 11 A. M., she became restless, tossing toand fro, requiring two or three attendants to keep her in bed. At 11.20 she had another convulsion, followed at short interval by another, a few minuters before 12 another, when the Doctors arrived and determined that something must be done and speedily; patient insensible, and os still *undilated*; agreed upon bilateral incision of cervix uteri; drew her to the edge of the bed, limbs drawn up and supported by Dr. N. and myself. Dr. Hammond proceded to divide with a probe pointed bistory, the undilated neck of the os in a lateral direction on each side, cutting towards the right and left acetabulum, and then *forcibly* dilating with finger, introduced a hook, caught the fœtus in the groin, (being a breech presentation) drew it down, and in a few minutes delivered her of a dead female fœtus about seven months. Patient still insensible and circulation very feeble. Placenta taken away entire; but uterus not contracting well, introduced the hand, removed a quantity of clot; inserted a piece of ice, put her to bed, and ordered feet kept warm, head cool, and flax seed mucilage kept constantly applied to her tongue, which was very much swollen from being bitten during convulsions, although efforts were made to prevent it by introduction of piece of wood and spoon between her teeth. To meet at 5 P. M., during which time she had no more convulsions; condition comparatively comfortable; drew off urine with catheter.

11th, 8½ A. M. Again used catheter; patient seems rational, and answers questions by nod or shake of the head, tongue being too much swollen to articulate.

5 P. M. Ordered ol. ricini, ℥i., ol. terebinthenæ, ʒi.; used catheter; circulation 100; has a more natural appearance.

12th, 8½ A. M. Quiet this morning; bowels well acted on by the oil; did not use catheter, urine having been voided.

5 P. M. Restless; pulse 112; ordered anodyne draught.

13th, 8½ A. M. Did not sleep well; complains of pain in breast, which are distended and hard; ordered light diet, and to rub breast with camphor soap liniment; mind entirely clear, but does not remember what had happened.

14th. Improving.

15. Restless; ordered 40 drops elixir opium.

16th and 17th. Doing well.

18th. Discharged.

*Remarks.*—The foregoing case has thus been minutely described, because, to my mind, it was an extraordinary and peculiarly interesting one, from the protracted and persistent unyielding, rigid os uteri, and from the novel, but successful mode adopted for its relief. "Under the head of difficult labor, may properly be considered all cases, where from any cause, the delivery is retarded or rendered dangerous."

In this case the delivery was not only retarded, but rendered imminently dangerous by the rigid and unyielding condition of the os uteri. The remedies, according to the best standard authors, are blood-letting, nauseants, the use of belladonna to the parts, and above all, anæsthetisc. Recently, it has been suggested, in slow dilatation of the os, to divide, to a limited extent, a portion of the circular fibres, but I believe has not met with much favor from the profession, because it is considered dangerous, and rendering the patient liable to rupture of the uterus. Opiates have been recommended when the labor is tedious; its advocates claiming that it quiets the contractions, thereby giving time for the circular fibres to dilate.

In the case under consideration, the patient had been nauseated and vomited frequently; was bled *copiously*, and kept under the influence of chloroform for several hours; all of which had *no* influence in *dilating* the os uteri. The belladonna was not applied, because the writer has never seen any good effects from its use; and this opinion has been corroborated by the experience of his personal friends in the profession. A full opiate would have been given, (as the patient was persuaded she was not full term) had it not been for the hyperæmia of the brain, which, to my mind, clearly contraindicated its use.

Here, then, we have a case in which all the remedies have been employed that have been recommended, where the os does not dilate,—all of which had *no* effect.

The patient in imminent danger of dying from convulsions, and this bilateral incision of the cervix uteri was performed, which not only arrested the convulsions—having removed the cause—but terminated successfully; the patient having not one threatening symptom, but a speedy recovery.

Not having seen this particular operation reported, I desire to impress upon the profession the facility with which it can be performed when *necessary*, and that too with a reasonable hope of success.

Dr. Flewellen reported a plan, extemporized by him some years ago, to accomplish forced delivery, which he thought would generally obviate the necessity for this unusual and dangerous operation of section of the cervix uteri.

It was in the case of a lady about seven months advanced, who, without any other apparent cause than that of pregnancy, was seized with obstinate and uncontrollable vomiting, which persisted for several days in spite of all efforts to arrest it, and until it became evident that she must die without the accomplishment of speedy premature delivery.

To effect this, a *toy* balloon of pure india rubber, was procurred, a portion of its wall cut away, a flexible catheter introduced through the apperture, and the india rubber bag drawn over the end, or laid in plaits as smoothly as possible the distance of several inches along the catheter, and tied around it so securely as to prevent the passage of fluid.

The catheter thus surmounted was passed through the cervical canal, the cervix resting about midway the bag.

The nozel of a suitable syringe was then adjusted to the external open end of the catheter, and cold water gradually forced through it—thus expanding the rubber bag into the form of an hour-glass—the constriction representing the part within the canal, and the bulbous parts the expanded extremities of the india rubber sac,—one being within the internal, and the other without the external os uteri.

Dilatation and speedy delivery were accomplished without bad results.

Dr. Powell discussed the subject at some length: stated that the operation, under many circumstances, was certainly justifiable; but the necessity for such interference should be determined by a thorough knowledge of the symptoms, which indicate that the labor, if left alone to nature, would jeopardize the safety of the mother; alluded to many of the circumstances or obstacles justifying and even requiring the operation. Unyielding rigidity of the cervix, in a healthy condition, may require surgical interference; but generally the knife will not be required, unless the extensibility of the fibres has been destroyed by disease, or become undilatable in consequence of cicatrices. The propriety of the operation in case reported by Dr. Holt, was clearly proven. He also stated, that the principle of Dr. Flewellen's mode of producing premature labor was old; but the plan, new, practical, and ingenious.

Dr. Thomas, of Savannah, said that the plan suggested by Dr. Flewellen was of considerable importance to the profession, in connection with the production of abortion. He then gave his views at some length on the case reported by Dr. Holt. He congratulated the Association on the prospect of a return to its legitimate work—the discussion of scientific subjects connected with the medical profession.

Dr. W. F. Westmoreland remarked that the case just reported by Dr. Holt, of Macon, Ga., was one of great interest; and as there had been, by members, some doubts expressed as to the propriety of the bilateral section of the os uteri, he felt that every member who had been called to treat cases requiring such mechanical interference, should give the Association the result of their experience.

Impressed with this duty, he now proposed to give a brief report of a case in which, in connection with Drs. L. H. Orme and J. F. Alexander, he performed the same operation.

The subject was a *primipara*, twenty-five years of age.

From Dr. Orme, who had charge of the case for the first six days of the labor, he obtained the following history of the case:—

On Saturday, the 25th November, 1866, labor commenced, and continued with more or less intensity until the following Wednesday, when the membranes were ruptured. There was no dilation of the os up to this time.

After the "waters" were discharged, the pains increased in intensity until they were almost unendurable. At no time, notwithstanding the most terrible pains, did the os dilate to more than the size of a Mexican dollar.

On Friday, the 7th day of the labor, he saw, with Drs. Orme and Alexander, the case for the first time. The patient, at that time, was greatly exhausted, a little delirious, with frequent and feeble pulse; the os was dilated to the size of a silver dollar, the edges thick and rigid, presenting a cartilaginous feel.

It resisted every effort at mechanical dilatation. Upon consultation, it was decided to make a bilateral section of the os uteri, and deliver with the forceps. He made the section with a pair of scissors, extending the incisions from an inch and a half to two inches on either side. It was not found practicable to deliver with the forceps, and craniotomy was then resorted to; and after considerable difficulty, delivery of a well formed child was finally accomplished. The patient did not rally, but continued to sink; and died in ten hours after the operation.

In this case, as in that reported by Dr. Holt, the woman would have certainly died with the child in utero, but for some mechanical interference.

The choice in such cases is between a section of the os and Cæsarian section.

It was unnecessary to discuss the relative dangers of the two operations to the mother. He did not regard the section of the os uteri, under such circumstances, as a formidable operation. That Dr. J. Marion Sims had demonstrated that complete section of the neck of the uterus could be made without inducing any unpleasant symptoms; that in sterility, the result of some forms of mechanical dysmenorrhœa his favorite plan of treatment was the bilateral section of the neck, extending to the internal os. While he admitted that there would be more risk in making a section of the gravid than the non-gravid uterus, still, Dr. Sims's operations upon this organ, has taught us that such operations are not so formidable as was once supposed.

The fear of wounding the peritoneum should not deter us, as the portion of the uterus incised corresponds to the neck in the non-gravid uterus, and has no peritoneal covering.

He suggested that four or five sections or incisions would perhaps give more space for the passage of the child's head than the bilateral section; that in the next operation of the kind that he was called on to perform, he should adopt this plan.

Dr. Charters, our worthy President, then yielded the chair to Dr. T. S. Powell, 1st Vice President, for the purpose of giving his views on the subject.

Dr. J. F. Alexander reported a singular case of a citizen of Atlanta, who, upon entering his room, began combing his whiskers, and thousands of sparks were emitted and fell to the floor; and farther, that when he would grasp a common glass tumbler with his hand, it would break in pieces.

Dr. A. W. Griggs, of West Point, was then conducted to the stand, and entertained the Association in an able and eminently creditable manner upon the subject of electrical forces as connected with intermittent fever.

Dr. J. T. Banks, of Griffin, then offered the following resolution, which was adopted:—

*Resolved*, That we hereby tender a vote of thanks to our late President, and request a copy of his address for the Association.

On motion, meeting adjourned to meet at 3 o'clock, P. M.

---

3 O'Clock, P. M.

Meeting was called to order by the president. Minutes read and adopted.

The following resolution was then offered by Dr. George M. McDowell of Barnesville, and adopted:—

*Resolved*, That a committee of three be appointed by the President, to prepare an address to the public, on the true relation of charletans and their nostrums to legitimate medicine, to report at next meeting. Committee—Drs. McDowell, Holt, and Crawford.

The following resolution was then introduced by Dr. W. F. Westmoreland, of Atlanta:—

*Resolved*, That a committee of five be appointed to revise the Constitution of the Medical Association of the State of

Georgia, to report at next meeting. Committee—Drs. W. F. Westmoreland, Griggs, Ray, Banks, and Myers.

The following resolution was introduced by Dr. A. W. Griggs, of West-Point, and adopted:—

*Resolved*, That a committee af five be appointed to present a report of the Medical Topography of the State of Georgia, to report at next meeting. Committee—Drs. Griggs, Thomas, Flewellen, Alexander and Ford.

Dr. A. W. Griggs introduced the following resoultion, which was adopted:—

*Resolved*, That a committee of five be appointed to report upon the medicinal properties and uses of the various unofficenal indigenous plants of the State of Georgia, and other states with which they may be familiar.' Committee—Drs. J. G. Westmoreland, Charters, Crawford, Geddings, and Hammond.

The following standing committees appointed at the last annual meeting were continued:—

Committee to prepare sketch of the life and character of those members of the Association who have died since the meeting of 1861;—Drs. Ford, Banks, and Logan.

Committee to examine prize essays—Drs. Banks, J. G. Westmoreland, Ford, Drewry, and O'Keefe.

Committee to memorialize the legislature or registration of births and deaths—Drs. Habersham, Westmoreland, and Word.

On motion of Dr. Edwin S. Ray, of Atlanta, the name of Dr. DeS. Ford, of Augusta, was inserted instead of Dr. F. O. Dannally, removed from the State.

On motion of Dr. W. F. Westmoreland, one hundred dollars was offered for three essays, fifty dollars for the first, thirty dollars for the second, and twenty dollars for the third; or medals amounting to the same, as the essayist may prefer.

On motion of Dr. Ray, of Atlanta, Dr. J. G. Thomas, of Savannah, was elected orator for the next annual meeting.

The following resolution was then introduced by Dr. J. T. Banks, of Griffin.

*Resolved*, That the thanks of this Association be tendered to the following Rail Roads of Georgia, which have kindly made concessions in favor of members of said Association: Georgia R. R., Central R. R., Atlanta and West-Point R. R., Macon and Western R. R., Savannah and Gulf R. R., Augusta and Savannah R. R.

On motion of Dr. Ray, Dr. Ford, of Augusta, was appointed chairman of the committee of arrangements, with the privilege of selecting his associates.

On motion of Dr. Myers, of Savannah, the report of the late treasurer was received, and ordered to be spread upon the minutes.

The following resolution was then offered by W. F. Westmoreland, and adopted:—

*Resolved*, That in the opinion of this Association, there is no breach of the Code of Medical Ethics which governs the profession, in physicians contracting with the owners or agents of plantations for the treatment of freedmen in their employ: *provided*, that in each city, county, or neighborhood, uniformity of charges be observed, and underbidding avoided.

Upon motion of Dr. Word, of Atlanta, the late Treasurer was called upon for a full report.

The following resolutions were introduced by Dr. J. N. Simmons, of Atlanta, and adopted:—

*Resolved*, That the members of the Association highly appreciate the cordial welcome they have received on the part of the city authorities of Griffin, and that their thanks are due and are hereby tendered to the citizens, and especially to the ladies, for their kind offices in contributing to the pleasures of this body during their session in this city, furnishing such entertainments as are ever agreeable, and which are esteemed as evidences of kind feeling and good will to the profession.

*Resolved*, That the thanks of this body are tendered to the Trustees of the M. E. Church, for the use of their lecture room for its deliberations.

On motion of Dr. Holt, of Macon, a vote of thanks was tendered to the Recording Secretary and Treasurer for the prompt and efficient manner in which they discharged their duties.

On motion, the proceedings of this meeting of the Association were ordered published in the Medical Journals of the State.

There being no further business, the meeting adjourned to its next annual meeting, in Augusta, on the second Wednesday in April, 1868.

L. H. ORME, *Rec. Sec.*

